# Multistep Model of Cervical Cancer: Participation of miRNAs and Coding Genes

**DOI:** 10.3390/ijms150915700

**Published:** 2014-09-04

**Authors:** Angelica Judith Granados López, Jesús Adrián López

**Affiliations:** 1 Laboratorio de microRNAs, Unidad Académica de Ciencias Biológicas, Universidad Autónoma de Zacatecas, Av. Preparatoria S/N, Zacatecas 98066, Mexico; E-Mail: agranadosjudith@gmail.com; 2 Área Académica de Ciencias Básicas, Doctorado de Ciencias Básicas, Universidad Autónoma de Zacatecas, Av. Preparatoria S/N, Campus II, Zacatecas 98066, Mexico

**Keywords:** miRNAs, cervical intraepithelial neoplasia (CIN), cervical cancer

## Abstract

Aberrant miRNA expression is well recognized as an important step in the development of cancer. Close to 70 microRNAs (miRNAs) have been implicated in cervical cancer up to now, nevertheless it is unknown if aberrant miRNA expression causes the onset of cervical cancer. One of the best ways to address this issue is through a multistep model of carcinogenesis. In the progression of cervical cancer there are three well-established steps to reach cancer that we used in the model proposed here. The first step of the model comprises the gene changes that occur in normal cells to be transformed into immortal cells (CIN 1), the second comprises immortal cell changes to tumorigenic cells (CIN 2), the third step includes cell changes to increase tumorigenic capacity (CIN 3), and the final step covers tumorigenic changes to carcinogenic cells. Altered miRNAs and their target genes are located in each one of the four steps of the multistep model of carcinogenesis. miRNA expression has shown discrepancies in different works; therefore, in this model we include miRNAs recording similar results in at least two studies. The present model is a useful insight into studying potential prognostic, diagnostic, and therapeutic miRNAs.

## 1. Introduction

Cervical cancer is one of the most frequent diseases in the world, and the second type of cancer that kills most women worldwide, with an estimated global incidence of 470,000 new cases and over 200,000 deaths per year [1]. One of the first events in cervical cancer development is the infection with human papilloma virus (HPV). HPV is associated with benign and malignant cervical lesions infecting mucosa and epithelial surfaces of the cervix. HPV replication occurs exclusively in squamous stratified epithelium such as the epidermis and mucous membranes [2,3] and is dependent on the cellular differentiation state and abundance of important proteins like transcriptional factors, polymerases, splicing factors, and an RNA processing machinery [3].

HPV infection results in the expression of viral proteins that change transitory normal cell functions such as proliferation and differentiation. Some HPVs, such as HPV16 and HPV18, are associated with oncogenesis and are therefore considered “high risk” (HR) viruses. HR viral E7 oncoprotein interacts with retinoblastoma (Rb) protein family members and permits G1 to S transition through the transcriptional factor E2F release from Rb, its regulatory protein [4]. Target promoter E2F binding activates transcription of several genes involved in proliferation [2]. In addition, HR-HPV E6 protein interacts with p53 and induces its degradation via ubiquinitation, resulting in p53-null phenotype abrogating apoptosis and cell cycle checkpoints [3,5]. Besides misregulation of coding genes, HR-HPV oncoprotein expression causes dysregulation of non-coding genes like microRNAs (miRNAs).

Cancer is a genetic complex pathology that involves coding gene and non-coding gene abnormalities [6]. Normal cellular proliferation is regulated by proto-oncogenes promoting proliferation and is balanced by their counterpart, tumor gene suppressors, that inhibit cellular proliferation. Mutations that increase proto-oncogen activity convert these genes into oncogenes leading to tumor cell proliferation. Normally these genes are growth factors, growth factor receptors, transductional signaling proteins, and DNA binding proteins [7]. On the other hand mutations that inactivate tumor gene suppressors liberate the genetic brake, and thereby potentiate tumor cell proliferation. However, for tumor progression, both of these genes have to be affected. Cellular proliferation is not an autonomic event; it obeys intercellular communication, ensuring normal tissue integrity. Examples of intercellular signals are contact inhibition and anchorage dependent growth, which are both hallmarks of normal cells [8]. Recently, an interchange of miRNAs by cells was reported, suggesting another way of communication [9,10]. With the discovery of miRNAs, the concepts of oncogenes and tumor gene suppressors have expanded. miRNAs regulating oncogenes are known as anti-oncomiRs, and miRNAs inhibiting tumor gene suppressors are known as oncomiRs [6,11]. One of the most important features about miRNAs is that they regulate several mRNA targets [12,13], permitting the analysis of several coding genes with just one miRNA [14].

MicroRNAs are noncoding regulatory RNAs 19–25 nucleotides (nt) in size that are produced by RNA polymerase II (pol II) and III (pol III) derived from transcripts of coding or noncoding genes. Many miRNAs are tissue-specific or differentiation-specific, and their temporal and lived expressions modulate gene expression at the post-transcriptional level by base pairing with complementary nucleotide sequences of target mRNAs [15,16]. Depending on the degree of sequence complementarity binding of miRNAs to target mRNA, they inhibit protein translation and/or degrade target mRNA [13].

Bioinformatics prediction shows that each miRNA targets more than 100 RNA transcripts and up to one-third of the total number of human mRNAs is regulated by these non-coding genes [17,18]. Therefore, the actions of miRNAs exert profound effects on gene expression in almost every biological process. Proliferation, anchorage independent growth, apoptosis, migration, and invasion are regulated by miRNAs [19]. In fact, restoration of miRNA expression or miRNA inhibition alters cellular processes [19,20]. Therefore, miRNAs are a powerful tool for gene therapy, prognosis, and diagnosis of malignant diseases.

miRNA expression affected by HPV specifically occurs as consequence of cervical cancer [21], some others are altered independently of HPV infection as a cause of cervical cancer. However, it is too soon to distinguish between the involvement of miRNAs as a consequence and/or a cause of cancer, but it is a fact that they orchestrate gene profile changes to induce carcinogenesis [22,23]. Since each miRNA reflects more than 100 coding genes regulated in cancer progression they are ideal genes to make a model of multistep carcinogenesis. Even though most of the actual papers compare normal *versus* carcinoma state and just a few have evaluated different neoplasia states in the cervical cancer process, with these data we propose a model to explain the cervical cancer progression based on miRNAs expression and their target coding genes.

## 2. Altered miRNAs Expression in Cervical Carcinomas

Aberrant miRNAs expression is well recognized as a marker for several carcinomas [24]. Tumor miRNAs expression has been evaluated by numerous techniques including microarrays, sequencing, northern blotting, cloning, and reverse transcription-polymerase chain reaction (RT-PCR) [25,26,27,28]. The results of these evaluations vary between research groups. The difference could be explained by the sample preparation, the methodology used, and/or the population, however, it is possible to find expression patterns independently of the variables mentioned before if the experimented changes in miRNAs expression are strong enough and constant at least in two different works. These two characteristics in miRNAs expression could give us an insight into studying and assigning miRNAs functions in cervical cancer. One of the first studies made in cervical cancer involving miRNAs was done in 2007. In this work, they sequenced 166 miRNAs in normal tissue, cell lines and tumor tissue. They found six miRNAs with differential expressions. Let-7b, let7-c, miR-23b, miR-143 and miR-196b were down-regulated in cell lines and tumor tissue compared with normal tissue whereas miR-21 was up-regulated [27]. Since then, a large number of studies has addressed the importance of microRNAs in cervical cancer. For example, 157 miRNAs were analyzed by RT-PCR in normal tissue and invasive squamous cervical cells resulting in 68 miRNAs over-expressed and two under-expressed in the cancer cells. Among these, the miRNAs that recorded the biggest increase were miR-9, miR-127, miR-133a, miR-133b, miR-145, miR-199a, miR-199b, miR-199s, and miR-214, while miR-149 and miR-203 showed the lowest expression [25]. A microarray analysis showed that miR-182, miR-183, and miR-210 were up-regulated and miR-128, miR-143, miR-145, and miR-195 were down-regulated in cervical carcinoma compared to normal tissue [26]. One hundred and seventy four miRNAs were cloned from normal tissue, cell lines, HPV-infected raft tissue and cervical cancer. In these experiments, miR-15b, miR-16, miR-146a, and miR-155 were over-expressed while miR-143, miR-145, and miR-128, were down-regulated [28]. Four novel miRNAs (miR-1273f, miR-1273g, miR-5095, and miR-5096) have been discovered while searching the fragile sites related to cervical cancer. These miRNAs were noticed in SiHa, HeLa, C33-A, and tumors but not in normal tissue [29]. Another study showed that miR-886-5p was increased in cervical squamous cell carcinomas (SCC) compared to normal adjacent tissue, while miR-10a*, miR-30a*, (the star strand or passenger strand is generally degraded from miRNA duplex in miRNA biogenesis) miR-302d, miR-346, miR-518b, and miR-610 were decreased [30]. The number of miRNAs involved in cervical cancer has increased importantly. Fifteen miRNAs (miR-7, miR-18a, miR-20a, miR-20b, miR-31, miR-93, miR-141, miR-142-5p, miR-146b, miR-189, miR-200a, miR-200b, miR-210, miR-224, and miR-429) and 17 miRNAs (miR-1, miR-10b, miR-99a, miR-99b, miR-100, miR-127, miR-140, miR-143, miR-145, miR-152, miR-195, miR-214, miR-218, miR-320, miR-368, miR-376a, and miR-497) were up- and down-regulated in cervical cancer compared to normal tissue, respectively [23,31]. Eighteen miRNAs were up-regulated (miR-10b, miR-15a, miR-16, miR-17, miR-20b, miR-21, miR-93, miR-106a, miR-106b, miR-130b, miR-146b-5p, miR-155, miR-185, miR-195, miR-339-5p, miR-625, miR-941, and miR-1224-5p) and 16 were down-regulated (miR-99a, miR-100, miR-125b, miR-139-5p, miR-139-3p, miR-145, miR-199a, miR-199b-5p, miR-149, miR-328, miR-375, miR-379, miR-381, miR-497, miR-574-3p, and miR-617) as well in SCC compared to normal tissue [32]. Table 1 lists all miRNAs with evident expression changes that have been reported in at least two different studies of cervical cancer. Constant gene expression changes in cervical cancer are important in order to be able to discover genes implicated in carcinogenesis. In this sense, the increase of chromosome 5p is seen in over 50% of advanced SCC, and Drosha, a miRNA processing protein, is localized in this region. Drosha transcript levels and expression were not elevated in pre-malignant cervical squamous intraepithelial lesions contrary to malignant lesions. miRNAs most significantly associated with Drosha over-expression are implicated in carcinogenesis, suggesting that they regulate fundamental processes in cancer progression. Interestingly, they reported that let-7, miR-15b, miR-21, miR-31, miR-107, miR-125-5p, miR-191, miR-200c, miR-203, and miR-330-3p were over-expressed, whereas miR-193b was under-expressed, implying selective miRNAs expression for cancer development [33]. Additionally, it has been shown that Drosha over-expression provides invasion and migration advantages in tumor cells [34].

**Table 1:** miRNAs with constant change in at least two experimental studies. Star strand (miRNA*) or passenger strand is generally degraded from miRNA duplex in miRNA biogenesis.

Name of miRNA	Expression Level	Technic	Type of Tissue	Reference
miR-1	Down	Microarray	Cancer	[23]
	Down	Cloning and Sequencing	Cancer	[35]
miR-7	Up	Microarray	Cancer	[23]
	Down	RT-PCR	Cancer	[36]
	Up	Cloning and Sequencing	Cancer	[35]
miR-9	Up	RT-PCR	Cancer	[25]
	Up	RT-PCR	CIN 2, 3 and cancer	[37]
miR-10a	Up	Microarray	CIN 1, 3 and Cancer	[38]
	Up	RT-PCR	CIN 2, 3 and Cancer	[37]
	Up	RT-PCR	Cancer	[39]
miR-10b	Down	Microarray	Cancer	[23]
	Up	Microarray	Cancer	[32]
	Down	Cloning and Sequencing	Cancer	[35]
miR-15a	Up	Microarray	Cancer	[32]
	Up	Microarray and RT-PCR	Cancer	[40]
miR-15b	Up	Cloning and Sequencing	Cancer	[28]
	Up	Microarray	Cancer	[33]
	Up	Microarray	Cancer	[34]
	Up	Microarray	CIN 2, 3 and Cancer	[22]
miR-16	Up	Cloning and Sequencing	Cancer	[28]
	Down	Microarray	CIN 1, 3 and Cancer	[38]
	Up	Microarray	Cancer	[32]
	Up	RT-PCR	CIN 1, 2, 3 and Cancer	[41]
miR-17-5p	Up	Microarray	Cancer	[32]
	Down	RT-PCR	Cancer	[37]
	Down	RT-PCR	Cancer	[42]
miR-19a/b	Up	Microarray	CIN 2, 3 and Cancer	[22]
	Up	RT-PCR	Cancer	[43]
miR-20a	Up	Microarray	Cancer	[23]
	Up	RT-PCR	Cancer	[44]
	Up	Microarray and RT-PCR	Cancer	[45]
	Up	RT-PCR	Cancer	[46]
miR-20b	Up	Microarray	Cancer	[23]
	Up	Microarray	Cancer	[32]
	Up	Microarray and RT-PCR	Cancer	[40]
	Up	RT-PCR	CIN 2, 3 and Cancer	[37]
miR-21	Up	Cloning	Cancer	[27]
	Up	Microarrays and RT-PCR	Cancer	[33]
	Up	Northern blot and Microarray	Cancer	[47]
	Up	Microarray	Cancer	[32]
	Up	Microarray	CIN 2, 3 and Cancer	[22]
miR-23b	Down	Cloning	Cancer	[27]
	Down	RT-PCR	Cancer	[48]
	Down	Microarray	CIN 2, 3 and Cancer	[22]
miR-26a	Down	Microarray	CIN 1, 3 and Cancer	[38]
	Down	Microarray and RT-PCR	Cancer	[45]
	Down	Microarray	CIN 2, 3 and Cancer	[22]
miR-27a	Down	Microarray and RT-PCR	CIN 1, 3 and Cancer	[38]
	Up	Microarray	CIN 2, 3 and Cancer	[22]
	Up	RT-PCR	CIN 1, 2, 3 and Cancer	[41]
miR-27b	Down	Microarray	CIN 2, 3 and Cancer	[22]
	Down	Microarray	Cancer	[45]
miR-29a	Down	Microarray and RT-PCR	CIN 2, 3 and Cancer	[49]
	Down	Microarray	CIN 1, 3 and Cancer	[38]
	Down	RT-PCR	CIN 1, 2, 3 and Cancer	[41]
	Up	Microarray	CIN 2, 3 and Cancer	[22]
miR-31	Up	Northern blot and Microarray	Cancer	[47]
	Up	Microarray and RT-PCR	Cancer	[33]
	Up	Microarray and Northern Blot	Cancer	[34]
	Up	Microarray	Cancer	[23]
	Up	Cloning and Sequencing	Cancer	[35]
miR-34a	Down	Northern Blot	Cancer	[21]
	Down	Microarray and RT-PCR	Cancer	[45]
	Up	Microarray	CIN 2, 3 and Cancer	[22]
	Down	RT-PCR	CIN 1, 2, 3 and Cancer	[50]
miR-92a	Up	Microarray and RT-PCR	CIN 2, 3 and Cancer	[49]
	Up	Microarray	CIN 2, 3 and Cancer	[22]
	Up	RT-PCR	CIN 1, 2, 3 and Cancer	[41]
miR-93	Up	Microarray	Cancer	[23]
	Up	Microarray	Cancer	[32]
	Up	Microarray	CIN 2, 3 and Cancer	[22]
	Up	RT-PCR	Cancer	[31]
miR-99a	Down	Microarrays	CIN 1, 3 and Cancer	[38]
	Down	Microarray and RT-PCR	CIN 2, 3 and Cancer	[49]
	Down	Microarray	Cancer	[23]
	Down	Microarray	Cancer	[32]
	Down	Microarray	CIN 2, 3 and Cancer	[22]
miR-99b	Down	Microarray	Cancer	[23]
	Down	Cloning and Sequencing	Cancer	[35]
miR-100	Down	RT-PCR	CIN 1, 2, 3 and Cancer	[51]
	Down	Microarray	Cancer	[32]
	Down	Microarray	Cancer	[23]
	Down	Microarray	CIN 2, 3 and Cancer	[22]
	Down	RT-PCR	CIN 1, 2, 3 and Cancer	[41]
miR-106b	Up	Microarray	Cancer	[32]
	Up	Microarray	CIN 2,3 and Cancer	[22]
	Up	Microarray and RT-PCR	Cancer	[40]
miR-125a-5p	Up	Microarray	Cancer	[34]
	Up	Microarray	CIN 2, 3 and Cancer	[22]
	Down	Microarray and RT-PCR	Cancer	[45]
miR-125b	Down	Microarray	Cancer	[32]
	Down	Microarray	CIN 2, 3 and Cancer	[22]
	Down	Microarray and RT-PCR	Cancer	[45]
	Down	Cloning and Sequencing	Cancer	[35]
miR-133a	Up	RT-PCR	Cancer	[25]
	Up	Microarray, *in situ* Hybridization and RT-PCR	CIN 2, 3 and Cancer	[52]
	Up	RT-PCR	Cancer	[25]
miR-133b	Up	RT-PCR	Cancer	[25]
	Up	Microarray, *in situ* Hybridization and RT-PCR	CIN 2, 3 and Cancer	[52]
	Up	RT-PCR	Cancer	[25]
miR-143	Down	Cloning and Northern Blot	Cancer	[28]
	Down	Microarrays, RT-PCR and Northern Blot	CIN 3 and Cancer	[26]
	Down	Microarrays	Cancer	[23]
	Down	Microarray and RT-PCR	Cancer	[45]
	Down	Microarray and RT-PCR	Cancer	[53]
	Down	Cloning and Sequencing	Cancer	[35]
	Down	Microarray	CIN 1, 3 and cancer	[38]
	Up	Microarray	CIN 2, 3 and Cancer	[22]
miR-145	Up	RT-PCR	Cancer	[25]
	Down	Microarray and Northern Blot	CIN 3 and Cancer	[26]
	Down	Cloning, Microarray and Northern Blot	Cancer	[28]
	Down	Microarray	Cancer	[32]
	Down	Microarray	Cancer	[23]
	Down	Microarray and RT-PCR	Cancer	[45]
	Down	RT-PCR	Cancer	[54]
	Down	Microarray	CIN 1, 3 and cancer	[38]
	Down	Microarray	CIN 2, 3 and Cancer	[22]
miR-146a	Up	Cloning and Northern Blot	Cancer	[28]
	Up	Microarray	CIN 2, 3 and Cancer	[22]
	Up	RT-PCR	Cancer	[55]
miR-146b-5p	Up	Microarray	Cancer	[23]
	Up	Microarray	Cancer	[32]
miR-155	Up	Cloning	Cancer	[28]
	Up	Microarray and RT-PCR	CIN 2, 3 and Cancer	[49]
	Up	Microarray	Cancer	[32]
	Up	Microarray	CIN 2, 3 and Cancer	[22]
	Up	Cloning and Sequencing	Cancer	[35]
miR-191	Down	Northern Blot and Microarray	Cancer	[47]
	Up	Microarray	Cancer	[34]
	Up	Microarray	CIN 2, 3 and Cancer	[22]
miR-193b	Down	RT-PCR and Microarray	Cancer	[33]
	Down	RT-PCR	CIN 2, 3 and Cancer	[37]
	Up	RT-PCR, Microarray and Northern Blot	CIN 3 and Cancer	[26]
miR-195	Down	RT-PCR, Microarray and Northern Blot	CIN 3 and Cancer	[26]
	Down	Microarray and RT-PCR	CIN 2, 3 and Cancer	[49]
	Down	Microarray	Cancer	[32]
	Down	Microarray	Cancer	[23]
	Down	Microarray	CIN 2, 3 and Cancer	[22]
miR-196b	Down	RT-PCR	Cancer	[56]
	Down	Cloning	Cancer	[27]
miR-199a	Up	RT-PCR	Cancer	[25]
	Down	Microarray	CIN 1, 3 and cancer	[38]
	Down	Microarray	Cancer	[32]
	Down	Microarray	CIN 2, 3 and Cancer	[22]
	Up	RT-PCR	Cancer	[25]
miR-200a	Up	Microarray	Cancer	[23]
	Up	RT-PCR	Cancer	[31]
miR-200a*	Up	Microarray	CIN 2, 3 and Cancer	[22]
	Up	Cloning and Sequencing	Cancer	[35]
miR-200c	Up	Microarray	Cancer	[34]
	Up	Microarray	CIN 2, 3 and Cancer	[22]
	Down	RT-PCR	Cancer	[45]
	Up	Microarray	Cancer	[23]
miR-203	Down	RT-PCR	Cancer	[28]
	Up	Microarray	Cancer	[33]
	Down	Microarrays	CIN 1, 3 and cancer	[38]
	Down	Microarray	CIN 2, 3 and Cancer	[22]
	Down	RT-PCR	Cancer	[57]
	Down	RT-PCR	Cancer	[46]
	Down	RT-PCR	CIN 2, 3 and Cancer	[37]
	Up	Cloning and Sequencing	Cancer	[35]
	Down	RT-PCR	Cancer	[25]
miR-205	Down	Microarray	CIN 1, 3 and cancer	[38]
	Up	RT-PCR	Cancer	[58]
	Up	RT-PCR	Cancer	[35]
	Up	RT-PCR, Microarray and Northern Blot	CIN 3 and Cancer	[26]
miR-210	Up	RT-PCR, Microarray and Northern Blot	CIN 3 and Cancer	[26]
	Up	Microarray	Cancer	[23]
	Down	Microarray	CIN 2, 3 and Cancer	[22]
miR-214	Down	Northern Blot and Microarray	Cancer	[47]
	Down	Microarray	Cancer	[23]
	Down	RT-PCR	Cancer	[59]
	Up	RT-PCR	Cancer	[25]
miR-218	Down	RT-PCR, Microarray and Northern Blot	CIN 3 and Cancer	[26]
	Down	Microarray	Cancer	[23]
	Down	RT-PCR	Cancer	[60]
	Down	Microarray	CIN 2, 3 and Cancer	[22]
	Down	RT-PCR	Cancer	[61]
	Down	RT-PCR	CIN 1, 2, 3 and Cancer	[62]
miR-224	Up	Microarray	Cancer	[23]
	Up	RT-PCR	Cancer	[63]
miR-375	Down	Microarray	Cancer	[32]
	Down	Microarrays and RT-PCR	CIN 2, 3 and Cancer	[49]
	Down	Microarray	CIN 2, 3 and Cancer	[22]
	Down	RT-PCR	CIN 2, 3 and Cancer	[64]
	Down	Microarray and RT-PCR	Cancer	[45]
miR-424	Down	RT-PCR	CIN 1, 2, 3 and Cancer	[65]
	Down	RT-PCR	Cancer	[66]
	Down	RT-PCR	Cancer	[37]
miR-497	Down	Microarray	Cancer	[23]
	Down	Microarray	Cancer	[32]
	Down	Microarray	CIN 2, 3 and Cancer	[22]

## 3. miRNAs Implicated in Cervical Cancer Progression

miRNA expression profiles have shown progressive expression changes between normal, cervical intraepithelial neoplasia (CIN) 1, 2, 3, and SCC. In a recent work, miR-29a, miR-99a, miR-195, and miR-375 were shown to be down-regulated in HPV16 CIN 2 and 3 *versus* normal tissue, and the expression continued diminishing in SCC. In contrast, miR-92a and miR-155 had an opposite expression pattern in CIN 2, 3 and SCC [49]. Later a constant and progressive reduction of miR-29a and miR-100 from CIN 1 to CIN 3 and cervical cancer was shown, while miR-16, miR-25, miR-27a, miR-92a, and miR-378 recorded an increased expression [41]. However, miR-375 participation in progression is not clear because its expression was decreased in CIN 2 and 3 compared to SCC but not between normal tissue and CIN 2 and 3, suggesting a participation in the latter stages of cancer development [64]. miR-375 expression in cervical cancer progression needs further work to elucidate its clear participation in carcinogenesis.

Other works have shown differential expression profiles between normal cervical tissue, CIN, and cervical cancer. A progressive expression reduction of miR-143, miR-145, and miR-218 was shown in CIN 3 toward cervical cancer [26,38]. In cervical cancer and HPV infected raft tissue from pre-neoplasic lesions *versus* non-infected raft tissue, an increased expression was found in miR-15, miR-146, and miR-424, while a down-regulation was seen for miR-143 and miR-145 [28].

A different study revealed that miR-26a, miR-29a, miR-99a, miR-199a, miR-203, and miR-513 were decreased in CIN 2, 3 and carcinoma while miR-10a, miR-132, miR-148a, miR-196a, miR-302b, miR-512-3p, and miR-522 were increased [38]. The analysis between SCCs, CIN 2, 3, and normal tissue showed 33 miRNAs with concordant differential expressions. Eighteen miRNAs were up-regulated (let-7i, miR-19b, miR-21, miR-25, miR-28-5p, miR-30e, miR-34a, miR-34b*, miR-92a, miR-92b, miR-106b, miR-146a, miR-181d, miR-200a*, miR-206, miR-338-5p, miR-592, and miR-595) and 15 miRNAs were down-regulated (miR-23b, miR-134, miR-149, miR-193b, miR-203, miR-210, miR-296-5p, miR-365, miR-370, miR-493, miR-572, miR-575, miR-617, miR-622, and miR-638) [22]. A progressive miR-129a-5p down-regulation from CIN 1 to 3 and cervical cancer *versus* normal tissue was recently shown [67].

Until now there are some issues regarding miRNAs profile changes in different studies. For example, while the Lee group [25] showed an increase, the Yang group [20] showed a reduction in miR-214 expression. An additional study reported a miR-214 expression increase [23]. miRNA profile changes should be taken with care because in some studies they are up-regulated and in others they are down-regulated (Table 1). It is also important to mention the unique gene expression of each patient.

## 4. miRNAs Regulated by HPV Oncoproteins

MicroRNAs misregulation in cervical cancer partly follows loss or gain of miRNA function after HPV integration [68,69]. Viral integration occurs mostly in transcriptionally active regions that include intron and/or exon sequences [70], affecting coding and non-coding genes. The studies so far have shown only a few miRNAs directly regulated by HPV oncoproteins. HPV L2 proteins are associated with a reduction of miR-125b expression and an increase of HPV DNA content. Recovering or reducing miR-125b expression, decreases or increases HPV DNA content, respectively [71]. L2 protein has not been classified as an oncoprotein but this new function could be the first evidence to rethink the classification of L2. MiR-125b levels could be crucial in people with HPV infection. Furthermore, it has been shown that miR-125b expression decreases in cervical cancer *versus* normal tissue [22,32]. The mechanisms leading to miR-125b reduction could be the result of DNA and histone methylation silencing [72] probably induced by HPV oncoproteins. Cells expressing HPV-16 E5 protein augment miR-146a expression while miR-324-5p and miR-203 diminish [73]. MiR-146a expression has been addressed as being important in cervical pathogenesis since it increases cell proliferation [28]. Single nucleotide polymorphism (SNPs) of miR-146a G allele significantly increases SCC risk [74,75] as the SNP alters miR-146a maturation [76]. MiR-218 is reduced by HR-HPV-E6-dependent expression [26]. Interestingly, miR-218 levels in patients with HR-HPV infection were lower than in those infected with low-risk or intermediate-risk HPV or in those who were HPV-free. Additionally, miR-218 levels were lower in high grade CIN than in those with low grade CIN [62]. Furthermore, the low serum levels of miR-218 in cervical cancer correlated with later stages of cervical cancer, adenocarcinoma, and lymphatic node metastasis [60]. Therefore, it appears that miR-218 down-regulation is involved in pathogenesis and progression of cervical cancer [62].

The actions of p53 are essential and more complex than it was thought, because it represses and/or activates coding and non-coding genes at the transcription level and during their biogenesis. P53 binds to several miRNA promoters to induce repression. Examples of this regulation are the repressions of miR-106b, miR-93, miR-25, and miR-17-5p, miR-18a, miR-19a, miR-20a, miR-19b-1, miR-92-1, and miR-106a, miR-18b, miR-20b, miR-19-2, and miR-92-2 [77,78].

HPV E6-expressing cells show a p53 null phenotype; in this context one can expect that all miRNAs regulated via p53 are going to be affected by E6. It has recently been reported that miR-34a is regulated by E6-dependent expression in cervical cancer [21]. Actually, it has been shown that pri-miR-34a decreases gradually according to cervical cancer progression. Additionally, a reduction was evident in CIN 1 compared to CIN 2 as it was also observed for CIN 2 *versus* CIN 3. It is noteworthy that miR-34a expression was lower in normal HPV positive than in normal HPV negative epithelium, showing an E6-dependent expression via p53 [50].

Remarkably, it was shown consensus sites for p53 in miR-23b promoter recording reduced levels of miR-23b in an E6 expression environment and uPA, one of its targets, shows an increased expression [48]. P53 expression seems to be determinant in cervical cancer progression, as it has been demonstrated for the transcriptional regulated miRNAs that could be activated by different signaling factors. MiR-34c expression is achieved by the p38 MAPK/MK2 pathway in p53-deficient cells [79].

On the other hand, the HPV-E7 protein affects the transcriptional function of E2F, thus it could be expected that this interference might affect miRNA expression. Binding sites for E2F1 and E2F3 were identified in the promoter of miR-15b. E2F induces transcription of miR-17-92, let-7a, let7-I, miR-15/16-2 and miR-106b [80,81,82]. MiR-15b expression was revealed to be highly correlated with the selected cell cycle E2F-induced genes CCNA2 (cyclin A2), CCNB1 (cyclin B1), MSH6 (mutS Homolog 6), and MCM7 (minichromosome maintenance complex component 7). HPV-E7 knockdown cell lines decrease miR-15b, MCM7, CCNB1 and CCNA2 expression, thereby inducing G1 arrest [83]. Another miRNA regulated by HPV is miR-203. It was recently reported that miR-203 is down-regulated in an HPV16-E7 dependent fashion. Furthermore, p63, a transcriptional regulator involved in carcinogenesis, is targeted by miR-203 via 3'-UTR, inhibiting protein production [84]. HPV oncoproteins alter the fine-tuning gene expression that regulates several cellular processes. Understanding this complex system will reveal the steps of the progression to cancer and suitable ways to block its advance.

## 5. Aberrant miRNAs Expression in Cellular Processes

Seven cellular processes have to be modified in the tumorigenic progression of cells: Evasion of proliferative signaling from growth suppressors, cell death resistance, support of replicative immortality, induction of angiogenesis, and activation of invasion and metastasis. Underlying these hallmarks is a genome instability, which generates a genetic diversity that accelerates the mechanism, similar to inflammation, to progress toward multiple hallmark functions. Normal tissues carefully control cellular processes that command entry into and progression through cell growth and division cycle, thereby ensuring homeostasis of cell number, invasion, and migration, thus maintaining normal tissue architecture and function [85]. Importantly, anti-oncomiRs and oncomiRs regulated cells are in charge of homeostasis. Normally, anti-oncomiRs hinder tumor processes by down-regulating oncogenes inhibiting the seven hallmarks of cancer. On the other hand oncomiRs favor tumoral processes by down-regulating tumor suppressor genes [6,85]. In cervical cancer we only have a few oncomiRs reported until now.

### 5.1. MicroRNAs Involved in Proliferation

miRNA expression loss is largely recognized as hallmark for cancer progression, increasing cell cycle continuation, proliferation, migration, invasion, and apoptosis inhibition [24,86]. Interestingly, miRNAs restoration reverts the excessive activity of the cellular processes mentioned above. MiR-7 [36], miR-17-5p [42], miR-34a [21], miR-34c [19], miR-124 [87], miR-100 [51,88], miR-143 [28,53], miR-125b [89], miR-145 [28], miR-155 [90,91], miR-196b [56], miR-214 [20,59,92], miR-218 [93], miR-203 [46,57], miR-302a, miR-302b, miR-302c, miR-302d, miR-367 [94], miR-375 [64,75] and miR-424 [66] mimic inhibition of proliferation and growth-independent anchorage of cervical cell lines. On the other hand miR-886-5p [30], miR-205 [58], miR-133b [52], miR-146a [28], miR-21 [95], miR-20a [44,46], miR-19a/b [43], and miR-9 [22] expression increases cervical cell line proliferation.

### 5.2. Cell Cycle and Apoptosis

Cellular proliferation is controlled by apoptosis and cell cycle progression. It is well documented that miRNAs can inhibit cell cycle phase transition. MiR-29a, miR-29b, miR-155 [49,90,91], miR-302a, miR-302b, miR-302c, miR-302d, miR-367, [94] miR-375, and miR-424 [66,75] inhibit G1/S transition in cervical cell lines. The transition from S to G2 phase is inhibited by miR-372 and miR-34c-3p over-expression in cervical cell lines [19,96]. The final G2 to M phase transition is arrested by a miR-100 mimic in cervical cell lines [92]. The great majority of miRNAs that arrest the cell cycle can also exert apoptosis; therefore, it is very difficult to recognize if inhibition of proliferation is achieved by cell cycle arrest or apoptosis induction. MiR-7 [36], miR-17-5p [42], miR-29a and miR-29b [49], miR-34a [21], miR-34c [19], miR-100 [51], miR-143 [53], miR-125b [89] and miR-424 [66] over-expression enhances apoptosis in SiHa, CaSki, HeLa and C33-A cells. Apoptosis induction by miR-34 family members’ restoration appears to be common in cervical cancer, as it has been shown for miR-34a and c. However, the 5p and 3p arms of miR-34c have different effects on apoptosis induction, as it was shown for miR-34c-3p that induced apoptosis but not miR-34c-5p in SiHa cells [19]. Interestingly, a study showed that anti-oncomiRs, miR-886-5p, and miR-182 expression reduced apoptosis in cervical cell lines [30,97]. Research has demonstrated that renewal of a single involved miRNA decided whether suppression or induction of apoptosis is sufficient to impede and/or favor cancer progression. Although tumors growth is an important issue in cancer development, the biggest drawback in health is metastasis. This cellular process is the least known at the moment, and miRNA equilibrium restoration has given us important insights into this field.

### 5.3. Migration and Invasion

To address the question of what role microRNAs play in cancer, several groups have proved that some miRNAs participate in the progression of cancer by evaluating their participation in cell migration and invasion. To this respect, it has been shown that miR-23b [48], miR-34a [98], miR-34c [19], miR-100 [88], miR-124 [87], miR-145 [99], miR-155 [90], miR-196b [56], miR-200a [100], miR-214 [92], miR-218 [61], miR-375 [64,75], and miR-424 [66] over-expression suppresses the ability of cervical cell lines to migrate and invade. Interestingly, the over-expression of miR-10a [39], miR-20a [44], miR-19a/b [43], miR-205 [58], and miR-9 [22] induced migration and invasion. Furthermore, several groups have found a correlation between lymph node tissue metastasis and miRNAs. Metastasis involves normal and tumor cell interaction, exposure to several and different microenvironments and the immune system, processes that are all not evaluated in cell cultures. That is why it is an important issue to have a correlation between miRNAs expression and metastasis. At this point in time, the list of miRNAs involved in cervical cancer metastasis is short, but with active study in this area, great advances are going to be made in the near future.

Importantly, a correlation exists between over-expression and/or under-expression of miRNAs and their respective expression in cancer and lymph node tissue metastasis. In this respect, the up-regulation of miR-1246, miR-20a, miR-224 [63], miR-2392, miR-3147, miR-3162-5p and miR-4484 in lymph node metastasis has been proven [45,46]. Furthermore, miR-224 expression has frequently been correlated with vascular invasion, advanced international federation of gynecology and obstetrics (FIGO) stage, positive HPV and shorter survival times [63]. On the other side, it was reported that miR-100, miR-125b, miR-143, miR-145, miR-199a-5p, and let-7c expression is involved with a decrease in lymph node metastasis [101]. Regarding miRNAs tissue specific expression, the investigation of Zhao *et al*. [46] showed that miR-20a increased expression, while this miRNA expression was decreased in the study by Huang *et al*. [101]. In the former, the samples were from squamous cell carcinomas whereas in the latter, the samples were from neuroendocrine small cell cervical carcinoma. Those differences must be taken into account during the development of diagnostic methods, the evaluation of prognosis and therapeutic applications.

## 6. Construction of a Multistep Model of Carcinogenesis by Expression of miRNAs and Their Targets

Based on the current research reported regarding the expression of miRNAs and their targets in cervical neoplasia’s progression to cancer, we propose a multistep model of carcinogenesis in cervical cancer composed of four steps: (1) the changes that a normal tissue suffers to be transformed into CIN 1; (2) the consecutive changes suffered in CIN 1 to achieve CIN 2; (3) the sequential changes acquired during CIN 2 toward CIN 3; and finally (4) the changes occurring in CIN 3 to reach cervical cancer. Although the information available up to now is limited, we are certain of the upcoming of new evidence and we expect that new knowledge can be added to the model proposed here to better understand the process of cervical carcinogenesis.

### 6.1. MicroRNAs Misregulated in Step 1

**Figure 1:**
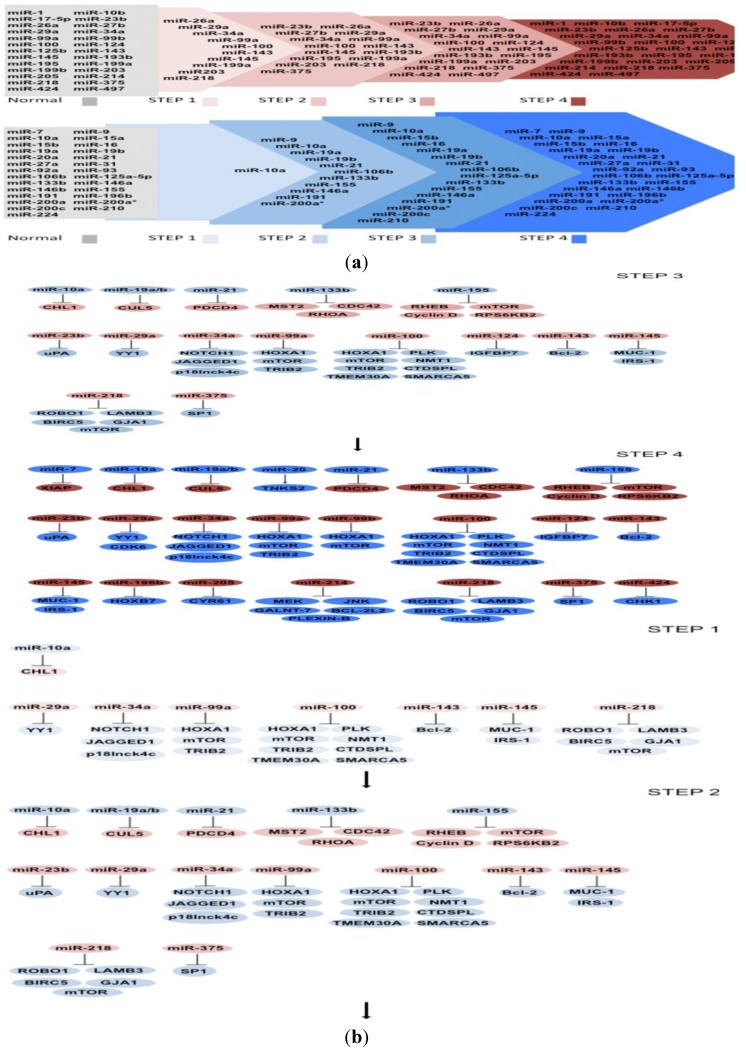
(**a**) MicroRNAs (miRNAs) implicated in cervical cancer progression. miRNAs are accommodated in steps 1, 2, 3 and 4 according to their expression. In the upper scheme, the arrow represents progressive miRNAs reduction. In the bottom scheme, the arrow represents progressive miRNAs increase; (**b**) miRNAs and their target coding genes implicated in cervical cancer progression. In the neoplasic stage, step 1, one oncomiR is up-regulated and its target is down-regulated. Additionally, in this step, seven anti-oncomiRs are down-regulated and twenty-three of their targets are up-regulated. In step 2, five additional oncomiRs are up-regulated and twelve of their targets are down-regulated. Likewise, two additional anti-oncomiRs are down-regulated and their targets are up-regulated. In step 3, only one additional anti-oncomiR is down-regulated and its target is up-regulated. In step 4, two additional onco-miRs are up-regulated with one target down-regulated and the other one up-regulated. In the same step, five additional anti-oncomiRs are down-regulated and ten of their targets are up-regulated, including CDK6 miR-29a target.

Even though the majority of miRNAs misregulated in cervical tissue have been found in later stages of cancer, some microRNA expression profiles have suggested their participation in cervical pathogenesis. The miRNAs down-regulation in CIN 1 reported up to now is due to miR-26a, miR-29a, miR-34a, miR-99a, miR-100, miR-143, miR-145, miR-199, miR-203, and miR-218 (Figure 1a). Most of them have already experimentally validated targets, except for miR-26a, miR-199a, and miR-203 (Figure 1b).

MiR-29a expression has been shown to be diminished during cervical pathogenesis, leading to apoptosis insensibility and uncontrolled cell cycles with increased Ying Yang (YY1) and CDK6 protein expression [102]. YY1 is an important transcription factor that inhibits apoptosis, and it has been shown to be over-expressed in cervical carcinomas [103]. On the other hand, CDK6 is a kinase that phosphorylates pRb releasing the transcriptional factor E2F [104]. It was shown that YY-1 protein expression began to increase from CIN 1 and 2, in contrast to CDK6 that was increased until SCC, Figure 1b. The over-expression of YY1 and CDK6 proteins inversely correlated with miR-29 expression in CIN 1, 2 and cervical cancer, respectively [102].

Another miRNA down-regulated in cervical cancer and HPV infected tissue is one of the members of miR-34 family, miR-34a. MiR-34a expression reduction is dependent on p53 regulation, therefore, expression reduction of other members of the miR34 family could also be attributable to p53 status, nevertheless, this has not been evaluated in cervical cancer. The pri-miR-34a shows a progressive reduction in CIN 1, 2, 3, and cervical carcinoma compared to normal tissue and shows an E6-dependent expression via p53 [50]. On the other hand, P18Ink4c is a CDK4/6 inhibitor that is increased in CIN 2 and carcinoma but not in normal cervix. Its participation in cancer progression seems essential because *de novo* infection of human keratinocyte-derived raft tissue by oncogenic HPV, increased p18Ink4c expression [105] contrary to miR-34a recording a reduction in cancer [21]. MiR-34a down-regulates p18Ink4c via 5'-UTR [105]. Additionally, miR-34a inhibits Jagged and Notch1 protein expression through 3'-UTR, affecting notch signaling [98]. Notch functions as a transcriptional factor implicated in controlling the expression of downstream genes associated with differentiation, cell fate specification, proliferation, apoptosis, adhesion, and angiogenesis [106]. A study showed that over-expression of the intracellular domain of Notch abolished miR-34a effect. Furthermore, urokinase-type plasminogen activator (uPA) is a serine protease that degrades extracellular matrix regulated through Notch signaling via miR-34a [98] and is considered to be involved directly in invasiveness and metastasis of cervical cell lines and probably participates in cervical cancer progression. As well as tribbles pseudokinase 2 (TRIB2) that belongs to a family that controls the specificity of the activation of mitogen-activated protein kinases (MAPK) [107], and its increased expression has been reported in several carcinomas [108,109], suggesting an oncogenic function. One member of the family miR-99, miR-99a, inhibits protein synthesis of TRIB2 via 3'-UTR [110]. This family is composed of miR-99a, miR-99b, and miR-100 [88]. The latter has shown a gradual reduction of expression in CIN 1, 2, 3 and cervical cancer relative to normal tissue. PLK1, a key mitotic checkpoint regulatory protein usually highly expressed in cervical cancer [111] is regulated at protein but not at mRNA level by miR-100 [51]. Even though PLK1 participates in G2/M phase check-point regulation to block cell progression induced by DNA damage, some tumor cells seem to override this check-point [112]. Until now, it is not known if this failure is a consequence of miR-100 reduction. MiR-99 family is an important issue in cervical carcinoma, as it has been shown for the three members that regulate mRNA and protein levels via 3'-UTR of the transcriptional factor homeobox A1 (HOXA1) and mTOR in HaCaT cells. Also, the phosphatase (CTD (carboxy-terminal domain, RNA polymerase II, polypeptide A) small phosphatase-like) (CTDSPL), enzyme *N*-myristoyltransferase 1 (NMT1), transmembrane protein 30A (TMEM30A), and chromatin remodeler SWI/SNF related, matrix associated, actin dependent regulator of chromatin, subfamily a, member 5 (SMARCA5) are targets of miR-100. The inhibition of these genes with siRNAs constrains proliferation, as also was achieved by over-expression of miR-100. In a similar way mTOR, HOXA1, CTDSPL, TMEM30A, and SMARCA5 siRNAs hinder HaCaT cell migration contrary to NMT1 siRNA experiments [88] that show specificity of migration capability. These results permit to conclude that miR-100 aberrant expression is important in cervical carcinogenesis.

In a similar manner, miR-143/145 clusters have tumor suppressive functions because they regulate numerous recognized oncogenes showing a diminished expression in CIN 1, 2, 3, and cervical cancer [26,28,38]. An inverse expression correlation of miR-143 and Bcl-2 is well documented in cervical cancer progression arguing an important issue in cancer development [38,113,114]. MiR-143 inhibits Bcl-2 mRNA and protein via 3'-UTR, abrogating proliferation and inducing apoptosis in HeLa cells as well as volume and tumor weight reduction in nude mice [53]. Additionally, it has been shown that IRS-1 and MUC-1 negatively regulate p53 [115,116] and miR-145, an effector of p53, inhibits IRS-1 and MUC-1 inhibiting migration and invasion but not cell proliferation. As it was mentioned before, E6 expression reduces p53 levels, causing miR-145 decrease [99].

In other studies, a progressive reduction of miR-218 levels in CIN 1, 2, 3 and cervical cancer has been shown [26,60,62]. Aberrant expression of miR-218 was more prominent in high-risk than intermediate-risk and low-risk HPV and HPV-free tissue [62]. Additionally, low serum levels of miR-218 in cervical cancer correlated with later stages, adenocarcinoma, and lymphatic node metastasis [60]. In addition, it was shown that pri-miR-218 SNPs are associated with cervical cancer risk, augmenting the potential of this microRNA as a good predictive and diagnostic marker [117]. The mRNAs modulated by miR-218 are baculoviral inhibitor of apoptosis repeat containing 5 (BIRC5), roundabout (ROBO1), connexin 43 (GJA1) and laminin 5 β3 (LAMB3) [26,61,102]. BIRC5 is a cancer-specific protein that has been shown to be up-regulated in cervical cancer and participates in apoptosis, proliferation, and angiogenesis [118,119]. ROBO 1 is repressed by epigenetic changes [120], however, the mRNA has been detected in SiHa cells [102]. Connexin 43 is a transmembrane protein that functions in the organization of cell-cell communication via gap junctions in multicellular organisms and is up-regulated in cancer [121,122]. Other studies have demonstrated that LAMB3, a protein preferentially expressed in the basal lamina of the epithelium was over-expressed in cervical cancer, and its expression induced migration and invasion in several cell lines [61,123,124]. MiR-218 inhibited cell migration and invasion via LAMB3 down-regulation but no effect was seen in cell proliferation [61], indicating a specific participation of these molecules in metastasis.

Rapamycin-insentive companion of mTOR (rictor) binds to mammalian target of Rapamycin (mTOR) to form the mTOR complex-2 (mTORC2). mTORC2 induces the phosphorylation of v-akt murine thymoma viral oncogene homolog (AKT) activating proliferation and cell survival. It has been shown that miR-218 counteracts proliferation and cell survival by the down-regulation of the protein rictor inhibiting phosphorylation of AKT and increasing caspase 3 and 8 activity in HeLa cells. Interestingly, Yamamoto *et al*. [61] did not observe an effect in cell proliferation in contrast to Li *et al*. [93]. The discrepancy between these two works could be based on the levels of miR-218 achieved in HeLa cells: while Li *et al*. [93] made a stable miRNA expression, Yamamoto *et al*. [61] induced a transitory expression of miR-218. Remarkably, miR-218 over-expression diminished weight and volume of tumors in nude mice, suggesting its study in human therapy [93].

In the same step of cervical cancer development, CIN 1, miR-10a is up-regulated and negatively modulates the cell adhesion molecule L1-like (CHL1). MiR-10a regulates the mRNA and protein expression via 3'-UTR of CHL1, promoting colony formation, migration, and invasion in HeLa and C-33A cells. Interestingly, miR-10a over-expression did not have any effect on cell viability of C-33A and HeLa cells showing cell process specificity. Furthermore, an increase and decrease of miR-10a and CHL1, respectively, was shown in cervical cancer tissue *versus* normal tissue [39]. MiR-10a also shows a progressive increase in CIN compared with cervical cancer [22,38]. According to the genes mistakenly expressed during CIN 1 and analyzed in this review, a group of changes is suggested that trigger a intricate restructuration of molecular and cellular events involved in check point regulation, cell signaling through AKT and MAPK, cell adhesion molecules, and epigenetic changes affecting proliferation, cell cycle, apoptosis, migration, and invasion (Figure 1b).

### 6.2. MicroRNAs Misregulated in Step 2

The changes achieved in CIN 1 continue to increase in CIN 2 along with 16 new unregulated miRNAs, six down-regulated and 10 up-regulated (Figure 1a). Importantly, until now not all miRNAs have targets validated experimentally in cervical cancer as can be seen in Figure 1a,b.

In this sense, only the anti-oncomiRs, miR-23b, miR-203 and miR-375; and the oncomiRs, miR-19a/b, miR-21, miR-133b, and miR-155 have experimentally validated targets, see Figure 1b. MiR-23b functions like a tumor suppressor miRNA because it is often down-regulated in HPV-associated cervical cancer contrary to one of its targets, (uPA) that is detected in cervical cancer but not in normal cervical tissues. MiR-23b diminishes uPA protein expression via interaction with its mRNA 3'-UTR. P53 absence via HR-HPV16-E6 oncoprotein was found to decrease the expression of miR-23b causing a uPA expression increase because the miR-23b promoter has a consensus p53-binding site [48].

Among the ways of gene expression regulation are the epigenetic changes that influence miRNAs expression, like DNA methylation, a hallmark for transcription silencing. A study demonstrated that the miR-203 promoter shows a great methylation status, conducive to a reduced level of mature miR-203 in cervical cancer *versus* normal tissue. Interestingly, miR-203 records a reduction in CIN 2 and 3 [22,38], causing an increase of vascular endothelial growth factor α (VEGFA). Furthermore, VEGFA was inhibited by miR-203 at the protein and mRNA level via 3'-UTR binding [57]. Additionally, p63 protein is increased upon HPV16-E7 expression in cells undergoing differentiation. This regulation has been inversely correlated with miR-203 expression, showing a mitogen-activated protein kinase-protein kinase C (MAPK-PKC) signaling dependence. It is possible that HPV16-E7 decreases miR-203 expression at the transcriptional and/or biogenesis level [84]. HPV-16 E7 interacts with HDACs [125,126] suggesting the possibility of an epigenetic silencing mechanism. Studies about miRNA processing suggest that HDAC1 is involved in pri-miRNA and/or pre-miRNA biogenesis. It has been reported that HDAC1 enhances miRNA processing via deacetylation of DiGeorge critical region gene 8 (DGCR8) [127]. Transcriptional networks are very important in cancer. The transcription factor Sp1 is inhibited by miR-375 at mRNA and protein level [75]. In cervical cancer, TGF-β1 transcriptional expression via HPV-E6/E7 protein expression through Sp1 has been reported [128]. Therefore, it is possible to expect a misregulation of genes with transcriptional sites for Sp1. The participation of Sp1 was evaluated in normal cervix *versus* carcinoma cells showing an inverse correlation with miR-375 [75]. Notably, miR-375 recorded a diminished expression in CIN 2, 3 and cervical cancer compared to normal tissue [64]. The network regulated by Sp1 and miR-375 could be fundamental in cervical carcinogenesis.

On the other hand, the oncomiR, miR-19a/b was found to inhibit cullin-5 (CUL5) also termed vasopressin-activated calcium mobilizing receptor (VACM-1) mRNA and protein via 3'-UTR. CUL5 was found to function as a tumor suppressor as it diminished cell growth and invasion in cervical carcinoma [43]. CUL5 participates in E3 ubiquitin ligase complexes targeting substrates for ubiquitin-dependent proteasome-mediated degradation [129,130], and it was shown to be involved in cellular proliferation and growth [131]. One can speculate that the target proteins for degradation by CUL5 are preferably those promoting cancer hallmarks. Likewise, an inverse expression between miR-21 and programmed cell death protein 4 (PDCD4) in invasive cervical cancer was shown. The function of PDCD4 in cell growth is probably given by blocking protein translation [132]. An increased expression of PDCD4 is recorded by miR-21 inhibition demonstrating a regulation via 3'-UTR in HeLa cells [95]. It is well documented that miR-21 is over-expressed in cervical pathologic tissue [22,25,27,28], acting as an oncomiR. MiR-21 over-expression is observed from CIN 2 to cancer compared to normal tissue either with negative or positive HPV infection [133]. In fact, an increase of miR-21 in exosomes of cervicovaginal lavage was shown in patients with cervical cancer and normal HPV positive subjects *versus* HPV negative patients [55]. Additionally, it was shown that miR-133b expression increased AKT and MAPKs (ERK1 and ERK2) phosphorylation, augmenting tumorigenesis. A gradual increase of miR-133b expression and AKT, ERK1, and ERK2 phosphorylation in CIN 2, 3 and cervical carcinoma were shown. AKT and ERK signaling are modulated via miR-133b down-regulation of mammalian sterile 20-like kinase 2 (MST2), cell division control protein 42 homolog (CDC42) and Ras homolog gene family member A (RHOA) at mRNA and protein level. MiR-133b increase and MST2 decrease, augments cell proliferation and colony formation in cervical cell lines [52].

P53 miRNA effectors inhibit cellular processes by altering their target expression. Some miRNAs and targets of p53 miRNA effectors regulate p53 levels. Over-expression of miR-155 increases p53 mRNA levels and inhibits mRNA cyclin D expression inhibiting CasKi cell proliferation, migration, and invasion [90]. Moreover, miR-155 down-regulates Ras homolog enriched in brain (RHEB), RAPTOR independent companion of MTOR, complex 2 (RICTOR) and ribosomal protein S6 kinase, 70kDa, polypeptide 2 (RPS6KB2) via 3'-UTR inhibiting mTOR-AKT signaling [91]. It seems that miR-155 acts like a tumor suppressor; nevertheless, its expression has been reported to be elevated in several works, see Table 1. It would be very interesting to evaluate the effect of miR-155 in other cell lines and/or mouse models of cervical cancer besides CaSki and HeLa cells. P53 regulation by miR-155 needs further investigation to have an accurate model of miRNA-P53 and P53-miRNA regulation. In CIN 2, the genes altered are involved in Notch signaling, transcription networks, proteasome system, protein translation, MAPK and AKT cell signaling, and cell cycle signaling giving advantages toward cervical carcinogenesis.

### 6.3. MicroRNAs Misregulated in Step 3

The changes observed in CIN 2, in addition to 3 and 5, lead to miRNAs down- and up-regulation, respectively, and are guidelines for the cellular and molecular changes toward CIN 3, see Figure 1a. We discuss the findings of miR-124 because its target, insulin-like growth factor BP7 (IGFBP7), and epigenetic methylation status have been recorded in CIN 3. MiR-124 is down-regulated by DNA methylation, resulting in IGFBP7 increase at the mRNA and protein level [87]. IGFBP7 has been implicated in cervical cancer, and it may influence the persistence of HR-HPV infection [134,135]. Furthermore miR-124 methylation is increased with cervical cancer progression showing a higher DNA methylation in CIN 3 and cervical cancer than in HR-HPV-positive tissues. Additionally, miR-124 restoration inhibits proliferation and migration implying that miR-124 methylation provides advantages for carcinogenesis [87]. The changes achieved in CIN 3 are related to the methylated environment and growth factors that could trigger down- and/or up-regulation of non-coding and coding genes permitting the acquisition of the cancer hallmarks that finally lead to the final step, cervical cancer.

### 6.4. MicroRNAs Misregulated in Step 4

More than 50 misregulated miRNAs have been reported in cervical neoplasias and cancers (Table 1). According to the data analyzed in this study, we have information of 19 and 16 miRNAs that are down- and up-regulated, respectively, in CIN 1, 2, 3 toward cervical cancer (Figure 1a). Despite the absence of information on the expression of 12 miRNAs in CINs, see Figure 1a, in this Step we discuss 7 miRNAs based on the knowledge of their validated targets in cervical cancer and their respective changes. The miR-17-5p, miR-125b-5p, miR-196b, miR-205, and miR-214 function as anti-oncomiRs while miR-7 and miR-20a work as oncomiRs. The decrease or increase of anti-oncomiRs and oncomiRs discussed in the last step are important in cervical cancer, however, their importance in cancer progression is still unknown because the reports mentioned here were only analyzed in normal tissue *versus* cervical cancer tissue, however, their expression was not evaluated in intermediate lesions.

An inverse correlation between miR-17-5p and the tumor protein P53-induced nuclear protein 1 (TP53INP1) has been shown. TP53INP1 responds and binds to p53, increasing p21 expression at the transcriptional level through promoter binding [136]. Notably, TP53INP1 was shown to be over-expressed in cervical cancer compared to normal tissue. Furthermore, the ectopic expression of TP53INP1 in cervical cell lines led to an increased proliferation. Additionally, it was shown that miR-17-5p down-regulated TP53INP1 at the mRNA and protein level via 3'-UTR binding, inhibiting proliferation and inducing apoptosis in cervical cell lines [42]. P21, BAX, PIG3 and MDM2 promoters are regulated by the interaction of TP53INP1 with p53 [136], therefore it is possible that some miRNA promoters regulated by P53 are regulated by the interaction with these transcription factors as well. It was shown that P53 binds to the promoter of miR-17-5p and suppresses its expression [137], but it is unknown if TP53INP1 participates in this regulation. It is important to mention that it is not known whether TP53INP1 tumor suppressor activity is dependent on p53 status, because p53 is down-regulated through HPV oncoprotein E6 expression in cervical cancer. TP53INP1 and p53 activity in miRNA promoter’s regulation needs further investigation, because transcriptional networks drive cell growth, cell division, and numerous cell signaling pathways that are regulated through the PI3K/AKT signaling pathway. This pathway has been shown to be on the one hand inhibited by miR-125b through down-regulation of mRNA and protein PIK3CD via 3'-UTR binding, leading to a decrease of protein kinase A (AKT) and mTOR phosphorylation and thereby inhibiting tumor growth and promoting apoptosis [89]. On the other hand miR-125b inhibits BAK1 protein synthesis via 3'-UTR binding mRNA, thereby inhibiting apoptosis and increasing tumor growth volume [138]. In the former study by Cui *et al*. [89], apoptosis was analyzed with a transitory miR-125b mimic in HeLa cells while in the later study by Wang *et al*. [138], the analysis was performed with transitory plasmids expressing pre-miR-125b. Another difference that could be noted was in reference to the controls. The work of Cui *et al*. [89] shows 2.2% of apoptotic cells, whereas that of Wang *et al*. [138] shows 7.5% apoptosis. Mice experiments performed by each group can not be compared because the work by Cui *et al*. [89] was done with HeLa cells expressing miR-125b, inhibiting tumor growth volume, while the work by Wang *et al*. [138] was done with HeLa cells expressing OCT4, increasing tumor growth volume. OCT4 over-expression induced the expression of numerous miRNAs such as miR-20a, miR-21, and miR-200c among others; therefore, the effect could be attributable to these miRNAs. OCT4 expression was observed in cervical cancer tissue but not in cervical cell lines. It should be noted that tissues are constituted of several types of cells other than tumorigenic. MiR-125b regulates numerous genes: oncogenes and tumor suppressor genes. A balanced inclination of oncogenes or tumor suppressor regulation by miR-125b should determine the effect. More studies are needed to elucidate the function of miR-125b, nevertheless strong evidence is presented regarding the regulation of PIK3CD and BAK1 by miR-125b.

Additional genes are important in the model such as those that participate in blood vessel formation, and those important to supply nutrients, growth factors, and oxygen to tumors. In this sense it was shown that VEGF transcription is generated by the transcriptional factor homeobox B7 (HOXB7), and that HOXB7 is regulated by miR-196b at protein and mRNA level via 3'-UTR binding [56], potentially affecting the genes regulated by these two coding genes. In the regulation displayed among miRNAs and their targets, it does not always end in a down- or up-regulation of genes, as it has been shown for miR-205 that binds to cysteine-rich, angiogenic inducer 61 (CYR61) and connective tissue growth factor (CTGF) mRNA. However, only CYR61 protein and mRNA were shown to be diminished by this miRNA. MiR-205 over-expression induces cell proliferation and migration in cervical cell lines. It was shown that miR-205 augments while CYR61 and CTGF mRNAs decrease in cervical cancer tissues, probably indicating that CTGF is regulated by other miRNA [58].

Some studies have addressed the importance of signaling proteins in cancer. For example, it was shown that the signaling through the trans-membrane receptor Plexin-B1 induced cell survival, proliferation, angiogenesis, invasion, and metastasis in cervical cancer [92]. Additionally, Plexin-B1 signaling was directly blocked by reduction of mRNA and protein via 3'-UTR binding by miR-214. Furthermore, miR-214 inhibited MEK3 and JNK1 at the mRNA and protein level, both genes that are involved in cell proliferation [20]. Another process related to cellular survival in cancer is the *O*-glycosylation that could be used to address tumor cells [139]. Nevertheless, the enzymes that participate in tumorigenesis are unknown. Recently, the enzyme UDP-*N*-Acetyl-α-d-galactosamine: Polypeptide *N*-acetilgalactosaminyltransferase 7 (GALNT-7) was recognized to increase proliferation, migration, and invasion in cervical cell lines recording a gene expression augment in cervical carcinoma. MiR-214 binds to the 3'-UTR of GALNT-7, inhibiting protein expression [140]. Additionally, miR-214 controls cell death through mRNA and protein down-regulation of anti-apoptotic proteins like Bcl-2l2, inducing the increment of Bax, Caspase 9, 8, and 3, triggering intrinsic/extrinsic apoptosis pathways [59].

The apoptosis trigger is dependent on the cell cycle checkpoint, and most of the carcinomas override this brake by gene modifications involved in these pathways. This is the case for miR-424 that is reduced in high-grade cervical neoplasia and is positively correlated with poor tumor differentiation, advanced clinical stage, and lymph node metastasis. The gene cell-cycle checkpoint kinase 1 (ChK1) is target of miR-424 and its inhibition decreases matrix metalloproteinase 9 expression. Cell cycle arrest in response to DNA damage is a normal activity of ChK1, but its expression was shown to be higher in high-grade carcinoma, suggesting its involvement in the pathogenesis of cancer [66].

There are only two oncomiRs identified with validated targets, but we are sure of the increase of miRNAs in this final step. MiR-7 and the X-linked inhibitor of apoptosis protein (XIAP) show a down- and over-expression, respectively, in tumors compared with normal tissues. The inhibitor of apoptosis protein XIAP functions like an E3 ubiquitin ligase targeting proteins for degradation by proteasome. Interestingly, miR-7 inhibits XIAP protein and mRNA via 3'-UTR in HeLa and C33-A cells [36]. The general mechanism of miRNA action is the decrease of gene expression, but by unknown mechanisms they could as well increase gene expression [141]. The proteins that interact with miRNA machinery biogenesis regulate miRNA maturation [142]. MiR-20a interacts with the 3'-UTR of tankynase 2 (TNKS2) up-regulating mRNA and protein expression. TNKS2 has the advantage of sustaining constant proliferation. TNKS2 is a new member of the human telomerase-associated poly (ADP-ribose) Polymerase (PARP) family and has been shown to be over-expressed in cervical cancer. TNKS2 protein binds to telomerase-binding protein TRF1 and protects the ends of linear chromosome. Therefore, ablation of TNKS2 and miR-20a inhibits colony formation, migration, and invasion. Remarkably, TNKS2 and miR-20a are high in cancer compared with normal tissue [44]. HPV16-E7 is an oncoprotein that causes chromosome alteration, therefore it could potentiate the effect of TNKS2 in carcinogenesis. In cervical carcinoma, the altered processes are related to migration, invasion, anchorage independent growth, cell cycle, and apoptosis through p53-interaction proteins, PI3K/ AKT cell signaling, growth, and angiogenesis factors, transmembrane receptors, intrinsic and extrinsic apoptosis, cell check points, apoptosis counteracts, chromosome ends, and cell cycle inhibitors.

## 7. Conclusions

In this review, we highlight cervical cancer associated miRNAs in CIN 1, 2, 3, and cancer as well as their targets. It is important to note that some of the miRNA targets in our model have not been directly evaluated in CIN. However, we assume that targets of altered miRNA are also going to be affected in carcinogenesis steps. However, this is not necessarily true, as it has been shown for miR-29a, YY1, and CDK6. MiR-29a is down-regulated and its targets are differentially regulated, while YY-1 is up-regulated in CIN 1, CDK6 is increased until SCC. Additionally, it should be noted that the expression of miRNAs that do not meet the criteria of having similar expressions in at least two studies are not included in this model. In the model that we propose, in the first step (CIN 1), miRNAs and their targets are involved in the regulation of check points, cell signaling through AKT and MAPK, cell adhesion molecules, and epigenetic changes affecting the hallmarks of cancer. Continuing with miRNA alterations in step 2 (CIN 2), cellular changes are achieved through Notch signaling, transcription networks, proteasome system, protein translation, MAPK cell signaling, and cell cycle signaling. Following is step 3, in which the changes achieved in CIN 3 are associated with epigenetic changes, although more studies are needed to further complement this step. And finally, in the cancer stage, the altered molecular processes are related to p53-interaction proteins, PI3K/AKT cell signaling, growth and angiogenesis factors, transmembrane receptor signaling, intrinsic and extrinsic apoptosis, cell checkpoints, apoptosis resistance, proteins participating in chromosome ends, and cell cycle inhibitors affecting cellular and molecular processes in carcinogenesis.

Along all the steps in cervical carcinogenesis the cell-signaling pathway with the most miRNAs implicated is the AKT pathway. Interestingly, miR-99 family, miR-125b, and miR-218 are diminished which is in contrast with the increased expression of miR-155 and miR-133b resulting in increased AKT phosphorylation. In cervical cancer progression, the AKT signaling is turned on, showing an important role in advantage acquisition of malignant transformation. Another disregulated cell pathway is MAPK cell signaling, which is a crucial factor in cancer progression. To this respect, an increased phosphorylation of MEK3/JNK and ERK1/2 by the reduced expression of miR-214 and increase of miR-133b has been shown. Further, Notch signaling is constantly activated to induce cell survival by the down-regulation of the regulators miR-34a and miR-23b that modulate several points of the signaling cascade.

The cell process most frequently found to be affected along neoplasia progression in this review is the cell cycle checkpoint. The G1 checkpoint is regulated through CDK6 and cyclin D expression by miR-29a and the network of miR-155-p53-miR-145 and p53-miR-34. CDK6 expression is up-regulated by the down-regulation of miR-29a, and cyclin D is regulated by miR-155, however, in several studies (Table 1), this miRNA is over-expressed, hence its function is unclear. Additionally, cyclin D is inhibited indirectly by miR-145 expression via p53-p21. On the other hand, P53 is mutated in 50% of carcinomas therefore miR-34a is down-regulated and its target p18Ink4c is increased in cervical cancer because the G1 check point is mutated most likely by the alteration of the feedback of miR-155-p53-miR-145, thereby overriding G1 checkpoint cell cycle. The G2-M checkpoint is overridden by the absence of its regulators, miR-100 and miR-424.

Another cell hallmark process of carcinogenesis is apoptosis. In this review, we discovered genes that were clearly involved in this important process. It is well known that p53 participates in G1 and G2-M checkpoints and that it can trigger apoptosis. Triggering or inhibiting apoptosis is fundamental for tumor survival. In this sense, miR-143 and miR-214 inhibit anti-apoptotic proteins, while miR-7 and miR-21 have the opposite function and inhibit apoptosis by the down-regulation of the pro-apoptotic proteins. Apoptosis resistance is achieved during the early steps, as the genes involved are deregulated.

Metastasis is the final process involved in cancer, and it is characterized by the formation of new tumors starting from the cells of the primary tumor. Tumor cells migrate with a set of different cell types to make an optimal niche to survive and grow. To this end, cell vessel formation is essential. The formation of new cell vessels is increased by the down-regulation of angiogenesis regulators miR-99 family, miR-196b, miR-203, and miR-205.

miRNAs and their targets are located sequentially in this cervical cancer multistep model (Figure 1a,b). Based on this initial model the miRNAs discussed here could be used to evaluate therapeutic, diagnostic, and prognostic applications in cervical cancer.
